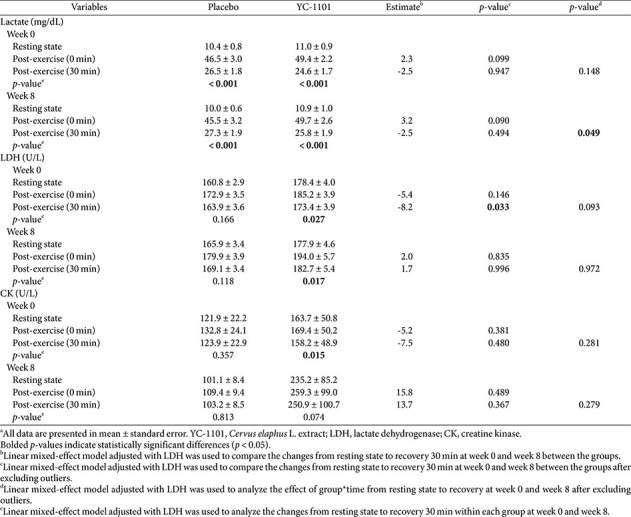# Efficacy and Safety of Enzyme-Derived Deer Velvet Extract Supplementation on Adults with Chronic Fatigue: a Randomized, Placebo-Controlled, Double-Blind Trial

**DOI:** 10.4014/jmb.2601.01055

**Published:** 2026-07-09

**Authors:** Parivash Jamrasi, Ji-won Seo, Yunho Sung, Donghyun Kim, Sanghyuk Han, Sowoon Kim, Chaewoon Kim, Xinxing Li, Hyun-Je Park, Aeri Song, Sinhwa Baek, Jeongho Jeong, Wook Song

**Affiliations:** 1 Institute on Aging, Seoul National University, Seoul 08826, Republic of Korea; 2 Health and Exercise Science Laboratory, Department of Physical Education, Seoul National University, Seoul 08826, Republic of Korea; 3 Department of Rehabilitation Medicine, Seoul National University College of Medicine, Seoul National University Bundang Hospital, Seongnam 13620, Republic of Korea; 4 College of Wushu and Dance, Shenyang Sport University, Shenyang 110102, China; 5 Institute of Sports Science, Department of Physical Education, Seoul National University, Seoul 08826, Republic of Korea; 6 Yuhan Care Co., Ltd., Yuhan Care R&D Center, Hwaseong 18469, Republic of Korea

**Keywords:** *Cervus elaphus* L, Enzyme-derived deer velvet extract, Anti-fatigue, Exercise performance, Chronic fatigue syndrome

## Abstract

Chronic Fatigue Syndrome (CFS) significantly impairs health-related quality of life in working-age populations, leading many individuals to use nutritional supplements for fatigue management. YC-1101, an enzymatically derived deer velvet extract, has demonstrated anti-fatigue potential in preclinical studies; however, clinical evidence in humans remains limited. This study aimed to evaluate the efficacy and safety of YC-1101 in adults with CFS. In an 8-week randomized controlled trial, 100 patients with CFS were assigned to either the YC-1101 or the placebo group. Subjective fatigue was assessed at baseline, at an interim point, and at the end of the intervention. Fatigue-related blood biomarkers and cardiorespiratory endurance were measured at baseline and postintervention. Compared with placebo, YC-1101 significantly improved Factor 1 (general and physical fatigue) of the Multidimensional Fatigue Inventory (MFI) at weeks 4 and 8, with a significant group-by-time interaction (*p* = 0.002), and improved the MFI items “I feel tired” and “I get tired easily” at both time points (group-by-time interaction *p* = 0.014 for each). YC-1101 also significantly improved fatigue-related motivation and functional interference on the Fatigue Severity Scale (FSS) at weeks 4 and 8, with significant group-by-time interactions (*p* = 0.040). After excluding outliers, the exercise distance to exhaustion was significantly greater in the YC-1101 group (*p* = 0.031), and lactate levels showed a significant group-by-time interaction at week 8 (*p* = 0.049). No significant differences in the safety outcomes were observed between the groups, and no adverse events were reported. Daily supplementation with YC-1101 for 8 weeks was safe, enhanced fatigue resistance, and improved exercise performance in adults with CFS, supporting its potential use in treating chronic fatigue.

## Introduction

As the average lifespan increases and society continues to age, public interest in health promotion is growing [1]. Health promotion aims to prevent diseases and maintain well-being by enhancing resistance to chronic and infectious diseases and promoting healthy lifestyles [2-4]. Recently, the functional food industry has focused on developing materials to relieve fatigue caused by modern lifestyles [5].

Fatigue is a subjective sensation encompassing physical, cognitive, and emotional features, characterized by a state of physical or mental exhaustion [6] and it can impair physiological function and quality of life [7]. Persistent fatigue lasting six months or longer, known as chronic fatigue syndrome (CFS), significantly affects health-related quality of life [8-10]. Individuals with CFS often experience energy deficiency, poor muscular endurance, and delayed recovery [11], and frequently use nutritional supplements to improve energy metabolism [11-13].

Deer antler extract (*Cervus elaphus* L., velvet antler) is a functional food ingredient traditionally used to restore energy and vitality [14]. Deer antler peptides exhibit various biological activities, including antioxidant, anti-inflammatory, anti-fatigue, and immunomodulatory effects [15, 16], and are widely used in East Asia [17]. Enzymatic extraction methods have been increasingly applied to enhance bioactive compounds compared to conventional hot water extraction [18, 19].

YC-1101, an enzymatically derived deer velvet extract, has demonstrated anti-fatigue and exercise performance-enhancing effects in animal studies. It improved exercise duration and reduced fatigue-related markers such as lactate dehydrogenase (LDH) and malondialdehyde in mouse models [20], and previous studies also reported enhanced endurance and improved metabolic recovery [21].

Recent studies have linked YC-1101 to fatigue-related mechanisms, including protection against mitochondrial stress in astrocytes and modulation of immune responses [22, 23]. Given that mitochondrial dysfunction and inflammatory cytokines are closely associated with fatigue [23], these findings support its potential role in fatigue management.

However, despite these promising findings, clinical evidence in humans remains limited. Therefore, this study aimed to evaluate the efficacy and safety of YC-1101 on fatigue, muscular endurance, and cardiorespiratory endurance in adults aged 30–59 with CFS. We hypothesized that YC-1101 supplementation would improve fatigue-related outcomes compared with placebo after 8 weeks.

## Materials and Methods

### Study Design

This study was designed as an 8-week randomized, double-blind, placebo-controlled trial with a parallel-group allocation. Study visits were conducted at four time points: screening (week -2), baseline (week 0), interim (week 4), and post-intervention (week 8). Eligible participants were assigned to a two-week run-in period before randomization and subsequently allocated to the YC-1101 or placebo group. Participants consumed the assigned supplements daily for 8 weeks and all outcome assessments were conducted according to the predefined visit schedule.

The sample size was estimated based on assumptions derived from previous clinical studies evaluating fatigue-related outcomes following dietary supplement intake [24-30]. Prior studies assumed between-group differences in changes of subjective fatigue scores ranging approximately from -2.2 to -8.0 and in biochemical fatigue markers ranging approximately from -9.4 to -161.1. Using these assumed values with a two-sided significance level of 5% and a statistical power of 80%, the required sample size for this study was calculated as 40 participants per group. Considering a potential drop-out rate of 20%, the target enrollment was set at 100 participants, with 50 assigned to each group.

### Participants' Eligibility Criteria

**Inclusion criteria.** Healthy adult participants aged 30-59 years were recruited based on the following criteria: 1) complained of chronic fatigue, 2) had a Fatigue Severity Scale (FSS) score of 28 or higher [6, 31]; and 3) agreed to participate in the study and provided written informed consent.

**Exclusion criteria.** Individuals were excluded if they were presented with: 1) inability to perform cycle ergometer testing; 2) participation in regular vigorous physical activity during the preceding 3-month period; 3) superior cardiorespiratory fitness (top 50% for age/sex) [32]; 4) Body mass Index below 18.5 kg/m^2^ or higher than 30.0 kg/m^2^; 5) alcohol intake exceeding 210 g/week (men) or 140 g/week (women); 6) current smoking or substance abuse; 7) insomnia (Insomnia severity index score ≥ 22); 8) history or current treatment for major chronic diseases (*e.g.*, cardiovascular, renal, hepatic, neurological disorders, diabetes, hypertension) or conditions requiring anticoagulants; 9) surgery within the previous 6 months; 10) consumption of herbal medicines or fatigue-related supplements in the past month; 11) regular intake of functional foods affecting fatigue in the past month; 12) known allergies to the product ingredients; 13) pregnancy, lactation, or pregnancy planning; 14) participation in other clinical trials within the past month; 15) inability to use digital devices (smartphone/computer); or 16) any other reason deemed unsuitable by the investigators.

### Randomization and Blinding

Eligible participants were allocated 1:1 to the supplement or placebo arm using computer-generated block randomization sequences. Allocation concealment was maintained by assigning sequential random numbers in the registration order. The participants, outcome assessors, and investigators remained blinded until completion of the statistical analyses. The supplements and placebo capsules were identical in appearance, taste, and smell.

### Interventions

The YC-1101 supplement was administered in capsule form. Each capsule contained 500 mg (240 mg YC-1101 as the active ingredient). YC-1101 is an enzymatically hydrolyzed deer velvet extract manufactured under standardized conditions. The deer antler (*Cervus elaphus*) used for YC-1101 was sourced from New Zealand and processed under controlled manufacturing conditions. The extract was prepared from *Cervus elaphus* antler by enzymatic digestion using flavourzyme at 50 ± 5°C for 12 h, followed by heat inactivation (90 ± 5°C for 20 min), filtration, concentration, sterilization, and freeze-drying.

For quality control and standardization, uracil and hypoxanthine were used as representative marker compounds. The contents of uracil and hypoxanthine in YC-1101 were 0.90 mg/g and 1.20 mg/g, respectively, as determined by high-performance liquid chromatography (HPLC). Representative HPLC chromatograms confirmed the presence of these marker compounds at retention times of approximately 6.7 min (uracil) and 14.8 min (hypoxanthine), ensuring batch-to-batch consistency of the extract (Fig. S1).

The selected dosage was based on preclinical efficacy data. An effective dose of 100 mg/kg in mice [20] was converted to a human equivalent dose using the U.S. Food and Drug Administration guidance, corresponding to approximately 480 mg/day, which was applied in this study.

Participants consumed two capsules once daily after meals, with sufficient water. All the supplements were stored at room temperature in a dry and dark environment. The placebo was also administered in capsule form and was identical to the YC-1101 supplement in terms of appearance, weight, and sensory characteristics. The control participants took two identical placebo capsules once daily after a meal with water, following the same consumption and storage conditions as the YC-1101 group. All participants were asked to maintain their habitual lifestyle while avoiding the intake of health supplements, high-dose vitamins, herbal injection therapies, and foods known to influence fatigue. The full compositions of YC-1101 and placebo capsules are listed in Table 1.

### Efficacy Measurements

During the intervention period, participants were instructed to maintain their usual diet and lifestyle. They were prohibited from consuming other health functional foods, herbal medicines, or fatigue-related supplements that could influence the study outcomes. To minimize variability, participants were asked to maintain consistent dietary patterns throughout the study and to record their dietary intake using the e-diary system. In addition, participants were instructed to follow similar dietary intake on the day prior to each visit and to abstain from alcohol and caffeine for at least 24 h before each assessment.

**Fig. 1:**
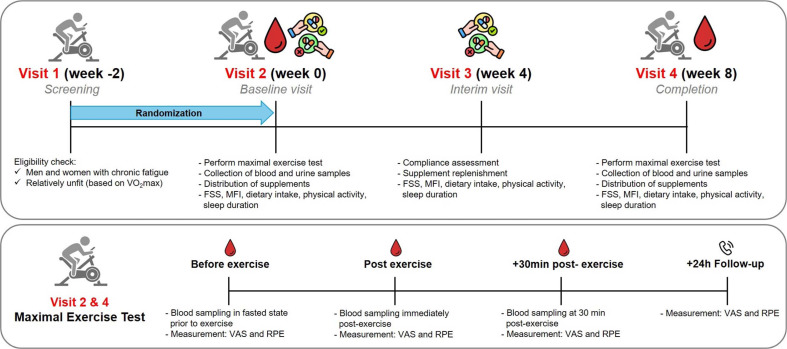
Study protocol; 8-week trial design comparing YC-1101 versus placebo supplementation in adults with chronic fatigue syndrome. YC-1101, Cervus elaphus L. extract; FSS, Fatigue severity scale; MFI, Multidimensional fatigue inventory; VAS, Visual analog scale; and RPE, Rating of perceived exertion.

In accordance with the predefined study schedule (Fig. 1), all participants completed a series of assessments comprising anthropometric measurements, compliance monitoring, dietary intake evaluation, assessments of physical activity and sleep duration, fatigue level measurement, and cardiorespiratory and muscular endurance testing.

**Anthropometric measures.** Height was assessed using a stadiometer with the participants in a standing position. BMI, skeletal muscle mass, and fat mass were measured using bioelectrical impedance analysis (InBody 770; InBody Co., Ltd., Republic of Korea). To ensure consistency, measurements were performed by the same personnel using the same equipment throughout the study.

**Compliance.** Compliance was assessed by counting the returned supplements and was calculated using the following equation:

*Compliance (%) = (number of capsules ingested / number of capsules prescribed) × 100*.

Daily supplement intake was monitored using a mobile application (E-diary, Biofood Co., Ltd., Republic of Korea).

**Dietary intake, physical activity and sleeping time.** To ensure that the clinical outcomes were not influenced by external lifestyle factors, dietary intake, physical activity, and sleep duration were monitored throughout the 8-week intervention. Participants recorded their dietary intake using the e-diary mobile application on three representative days between visits, which included two weekdays and one weekend. The Recommended Food Score (RFS) was used to evaluate dietary quality (Collins *et al*., 2015). Physical activity levels were assessed using the Global Physical Activity Questionnaire and total sleep duration was self-reported [33].

**Fatigue level.** Subjective fatigue was evaluated using the FSS and the Multidimensional Fatigue Inventory (MFI) [34]. Additionally, a visual analog scale (VAS) was used. To evaluate recovery from exercise-induced fatigue, VAS scores were collected at four time points: pre-exercise, immediately post-exercise, 30 min post-exercise, and 24 h post-exercise.

**Cardiorespiratory and muscle endurance.** Exercise performance was assessed using a cycle ergometer (Corival CPET, LODE, The Netherlands) according to a modified Åstrand protocol [35]. The participants rested for 30 min prior to the test. After a 1-min warm-up (0.5 kp), the workload was increased by 30 W (0.5 kp) every 2 min. The participants maintained a cycling cadence of 60–70 rpm throughout the test. Exercise testing was discontinued when the participants satisfied any of the following criteria: (1) inability to maintain at least 60 rpm for > 10 s, (2) Rating of Perceived Exertion (RPE) 17, or (3) exhaustion. Outcome measures included the total time to exhaustion, distance, VO_2_max, HRmax, and RPE. Blood biomarkers (lactate, LDH, creatine kinase [36]) were analyzed at baseline (pre-exercise), immediately after exercise, and 30 min after recovery.

### Safety Measures

**Vital signs.** Blood pressure, heart rate, and body temperature were measured after a 10-min resting period at each study visit.

**Clinical laboratory tests.** Hematological tests (WBC, RBC, Hb, Hct, PLT, MCV, MCH, MCHC, and differential blood count), blood chemical tests (ALT, AST, ALP, BUN, creatinine, total bilirubin, uric acid, total protein, albumin, and glucose), and urinalysis (pH, protein, glucose, ketone, urobilinogen, bilirubin, nitrite, and specific gravity) were performed before exercise. Blood samples were collected after participants fasted for 12h.

**Adverse events.** The safety was monitored continuously. The participants were encouraged to report any adverse symptoms, which were subsequently verified by the investigators during the interviews.

### Statistical Analyses

All statistical computations were performed using SAS software (version 9.4; SAS Institute, USA), employing parametric methods based on the data normality assumption. Analyses were performed using an intention-to-treat. Baseline demographic characteristics and compliance rates were compared using independent t-tests (continuous) or chi-squared tests (categorical). For the efficacy endpoints (including fatigue scores, cardiorespiratory fitness metrics, and muscle endurance biomarkers), a linear mixed-effects model was applied, incorporating group, time, and group-by-time interactions as fixed effects, with the subject as a random effect. Baseline LDH levels were included as covariates to adjust for baseline imbalance between groups and to control for their potential confounding effect on fatigue-related outcomes. Outliers in continuous efficacy variables were defined as values exceeding 1.5 × the interquartile range (IQR) and were identified for each variable at each time point. These values were excluded prior to the main analysis to minimize the influence of extreme observations on the results. The procedure was applied consistently across groups. Safety data were analyzed using linear mixed-effects models or appropriate exact tests (Fisher’s or McNemar’s test). Data are expressed as mean ± standard error or estimates of the difference in changes between groups, with statistical significance defined as *p* < 0.05.

### Ethics Statement

This study was approved by the Institutional Review Board of Seoul National University (IRB No. 2307/004-007) and adhered to the principles outlined in the Declaration of Helsinki. All participants provided written informed consent before enrolment in the study.

## Results

### Participants

**Fig. 2:**
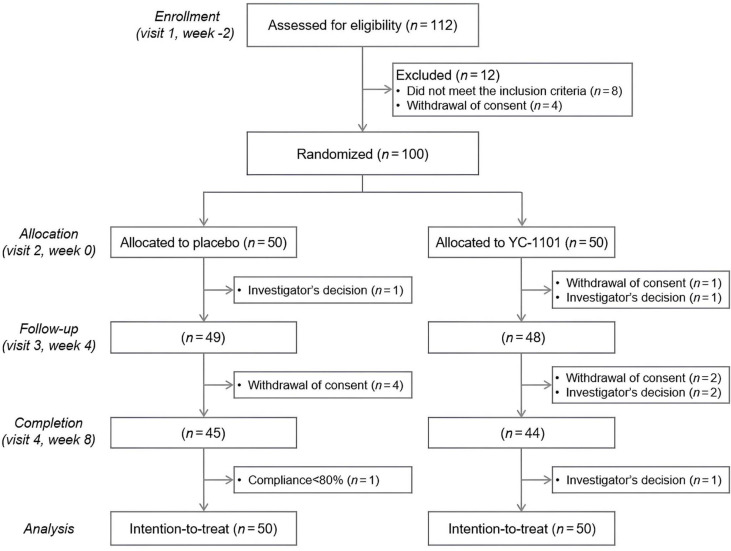
CONSORT flow diagram of study participants.

Of the 112 individuals screened, 100 eligible participants were equally randomized into placebo (n = 50) and YC-1101 (n = 50) groups. During the study period, five participants in the placebo group (four withdrew consent and one due to the investigator’s decision) and six participants in the YC-1101 group (three withdrew consent and three due to the investigator's decision) discontinued participation. All randomized participants were included in the ITT analysis and 89 completed the 8-week intervention (Fig. 2).

The mean age of participants was 43.9 ± 1.2 years in the placebo group and 43.8 ± 1.3 years in the YC-1101 group. Baseline FSS scores were 42.0 ± 1.1 in the placebo group and 42.5 ± 1.1 in the YC-1101 group. No significant differences in baseline characteristics were observed between the two groups, except for blood LDH levels (Table 2).

### Compliance with Supplements

Overall compliance with supplements exceeded 98% in both groups (placebo: 98.5 ± 0.8%; YC-1101: 98.5 ± 0.7%), with no significant difference between the groups (*p* = 0.996).

### Dietary Intake, Physical Activity, and Sleep Duration

As shown in Supplementary Table S1, there were no significant inter-group differences at the end of the study in total energy intake (*p* = 0.202) or nutrient consumption (carbohydrates, *p* = 0.056; protein, *p* = 0.564; fat, *p* = 0.903; sodium, *p* = 0.748). Additionally, physical activity levels (*p* = 0.204) and sleep duration (*p* = 0.700) remained consistent between the YC-1101 and placebo groups.

### Fatigue Level

The MFI results are summarized in Table 3. Factor 1, representing general and physical fatigue, comprised the sum of six items (Items 1, 5, 12, 14, 16, and 20). Compared with the placebo group, the YC-1101 group showed a significant improvement at week 4, with the effect maintained at week 8 (week 4: β = -2.8, *p* < 0.001; week 8: β = -2.1, *p* = 0.011), with a significant group-by-time interaction over the 8-week intervention period (*p* = 0.002). Among the individual items of Factor 1, item 5 (“I feel tired”) showed significant improvements at week 4, with the effects maintained at week 8 (week 4: β = -0.7, *p* = 0.007; week 8: β = -0.6, *p* = 0.023). For item 16 (“I tire easily”), fatigue significantly decreased at both time points (week 4: β = -0.6, *p* = 0.005; week 8: β = -0.5, *p* = 0.049), with a significant group-by-time interactions over the 8-week intervention period were identified for both items (*p* = 0.014 for each).

The FSS results are listed in Table 4. Compared with the placebo group, the YC-1101 group showed a significant reduction in item 1 of the FSS (“My motivation is lower when I am fatigued”) at week 4, and this effect was sustained through week 8 (week 4: β = -0.5, *p* = 0.038; week 8: β = -0.5, *p* = 0.022), with a significant group-by-time interaction over the 8-week intervention period (*p* = 0.040). Item 7 (“Fatigue interferes with carrying out certain duties and responsibilities”) showed a trend toward reduction at week 4, followed by a significant reduction at week 8 in the YC-1101 group compared with the placebo group (week 4: β = -0.5, *p* = 0.051; week 8: β = -0.6, *p* = 0.018). A significant group-by-time interaction over the 8 weeks was also observed (*p* = 0.040).

### Cardiorespiratory and Muscle Endurance

**Fig. 3:**
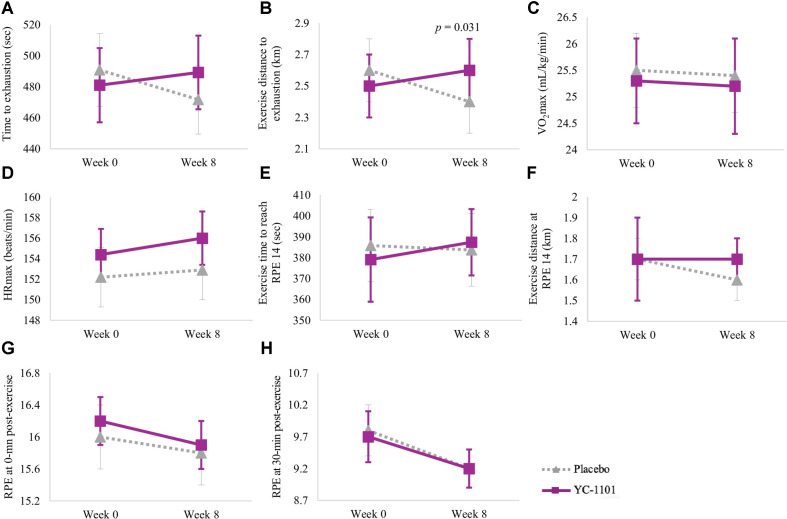
Changes in cardiorespiratory and muscle endurance. Changes over 8 weeks in the placebo and YC-1101 groups are illustrated in the following panels: (**A**) Time to exhaustion (sec). (**B**) Exercise distance to exhaustion (km). (**C**) VO_2_max (mL/kg/min). (**D**) HRmax (beats/min). (**E**) Exercise time to reach RPE scale 14 (sec) (**F**) Exercise distance at RPE scale 14 (km). (**G**) RPE score at 0-min Post-exercise. (**H**) RPE score at 30-min Post-exercise. All data are presented in mean ± standard error. Significant p-value represent the group-by-time interaction effects from a linear mixed-effects model after excluding outliers (*p* < 0.05).

Following the exclusion of outliers, the YC-1101 group demonstrated a significantly greater exercise distance to exhaustion compared with the placebo group (β = 0.1, *p* = 0.031; Fig. 3). In contrast, no significant between-group differences were observed in the time to exhaustion, VO_2_max, HRmax, RPE, or VAS scores.

### Blood Biomarkers

After excluding outliers, lactate levels exhibited a significant group-by-time interaction at week 8 in the YC-1101 group relative to the placebo group (*p* = 0.049; Table 5), whereas no other biochemical parameters showed significant differences.

### Safety Measures

**Vital signs.** No significant differences were observed between groups in terms of SBP, DBP, pulse rate, or body temperature.

**Clinical laboratory tests.** No significant between-group differences were observed in the hematological tests, blood chemical tests (Table S2), or urinalysis (Table S3). Although a statistically significant within-group change in WBC (week 0:5.3 ± 0.2 × 10^3^/μL; week 8:5.6 ± 0.2 × 10^3^/μL; *p* = 0.037) and eosinophil (week 0:3.1 ± 0.5%; week 8:2.6 ± 0.3%; *p* = 0.013) levels was observed in the YC-1101 group, the values remained within the normal reference range and were not considered clinically meaningful.

**Adverse events.** No serious adverse events related to the supplements were reported in either group.

## Discussion

This randomized, double-blind, placebo-controlled study is the first trial to evaluate the effects and safety of YC-1101 on fatigue as well as muscle and cardiorespiratory endurance in adults aged 30–59 years with CFS. Daily intake of 480 mg YC-1101 for 8 weeks demonstrated both efficacy and safety in reducing fatigue. Significant improvements were observed in MFI, FSS, muscle endurance, and fatigue-related metabolites in the YC-1101 group compared to the placebo group. These findings indicate that an increased distance to exhaustion corresponds with physiological responses during fatigue-inducing exercise, highlighting the greater resistance to exercise-induced fatigue in the YC-1101 group and reinforcing the observed improvements in fatigue.

YC-1101 has been reported to exhibit multiple biological actions that may contribute to the reduction of fatigue and cellular protection. Previous studies suggest that it may activate the Nrf2-Keap1 signaling pathway, enhance antioxidant enzyme expression, and support muscle recovery [20]. Additionally, previous studies have reported that YC-1101 may enhance natural killer cell activity and splenocyte proliferation in immunosuppressed mouse models [23], indicating its immunomodulatory potential. A recent study suggests that YC-1101 may protect astrocytic mitochondria from oxidative stress, thereby maintaining neuronal energy metabolism. By preserving mitochondrial integrity and sustaining neural energy balance, YC-1101 may potentially alleviate central fatigue and protect against neurodegenerative processes [22].

Given that central fatigue is closely intertwined with the generalized muscle fatigue characteristic of the CFS, the present study’s findings are consistent: individuals experienced improvements in general and physical fatigue, as well as muscular endurance, even under physical fatigue conditions. This aligns with previous descriptions of CFS, in which abnormal muscle fatigue can arise even after minimal activity [37]. Although no significant between-group differences were observed in the total MFI and FSS scores, significant improvements were observed in specific subdomains, particularly those related to general and physical fatigue. These domain-specific improvements may represent more sensitive indicators of intervention effects than total scores. In addition, while some outcomes showed borderline statistical significance, these findings were generally consistent across related measures, supporting a potential beneficial effect of YC-1101 rather than isolated results. Nevertheless, these findings should be interpreted with appropriate caution. Notably, fatigue is a multidimensional construct rather than a single concept [38, 39], and significant improvements were observed in the MFI Factor 1 (general and physical fatigue), which typically increased sharply after the 40s and ranked among the top fatigue-related subdomains up to the age of 75 years (Table 3) [34]. This subdomain also demonstrates high internal consistency, with Cronbach’s alpha coefficients of 0.84 (general fatigue) and 0.78 (physical fatigue), compared with 0.52–0.67 for other subdomains, indicating strong reliability and clinical relevance [40]. Although the FSS total scores showed only a decreasing trend compared to the controls, significant improvements and trends were observed in items related to motivation reduction, work-related impact, and recognition of fatigue as a problem (Table 4). Collectively, these findings suggest that YC-1101 may exert particularly positive effects on general and physical fatigue rather than on overall fatigue.

Consistent with the findings reported by Park *et al*. regarding the anti-fatigue effects of YC-1101, which demonstrated increased endurance and reduced lactate accumulation in C2C12 cell line and forced swimming mouse models [20], improvements in fatigue under physical stress were observed in this study. Despite all participants cycling at a fixed speed of 60–70 rpm, the intervention group showed a significantly greater exercise distance to exhaustion than the controls (Fig. 3B; *p* = 0.031). This indicates improved resistance to fatigue and aligns with previous human studies that examined the effects of anti-fatigue supplementation [6, 41, 42]. Notably, this significance was observed following the systematic exclusion of outliers, defined as values exceeding 1.5 times the IQR. This pre-specified approach was applied consistently across both groups to account for the high inter-individual variability characteristic of CFS. By minimizing the influence of extreme biological observations, these results more accurately reflect the general treatment effect of YC-1101 on exercise performance.

A previous animal study demonstrated the anti-fatigue effects of YC-1101 by showing reduced serum lactate and LDH levels during forced-swimming tests [20]. Consistent with this, favorable trends in exercise-induced muscle fatigue markers were observed in this study, with lactate levels notably improving following YC-1101 supplementation. Although a statistically significant difference in LDH levels was observed between groups, all values remained within the normal reference range, suggesting that the difference may not be clinically meaningful. Since lactate accumulation during exercise contributes to fatigue by increasing the hydrogen ion concentration and inducing muscle acidosis [43], reducing lactate levels is considered an important mechanism for improving recovery. After 8 weeks, the YC-1101 group showed significantly lower lactate levels at 30 min post-exercise (Table 5), indicating improved recovery under fatigue conditions. This finding suggests that YC-1101 may help slow the increase in fatigue-related metabolites and enhance their clearance in humans [42].

Despite these promising findings, this study has several limitations. First, the sample included more women than men, which may limit generalizability, although the sex ratio was balanced between the groups. Second, the study focused on adults aged 30–59, restricting its applicability to other populations. While diet and exercise were controlled for, other lifestyle differences could have influenced the outcomes, and potential confounders such as baseline LDH differences were identified and adjusted. Third, outliers were excluded to prevent extreme deviations from skewing the mean treatment effect. While we recognize that such values may reflect true biological variability, their inclusion significantly increased variance, potentially masking the central tendency of the data. Consequently, focusing on the most representative distribution allowed for a more robust and conservative estimation of YC-1101’s efficacy across the broader study population. To strengthen these conclusions, future studies should involve larger, more diverse populations and more comprehensive control of confounding variables. Such research will provide deeper insights into the efficacy and safety of YC-1101 supplementation and its potential role in fatigue mitigation.

In conclusion, an 8-week daily supplementation with 480 mg YC-1101 enhanced fatigue resistance and exercise performance in adults with CFS. Its safety was supported by assessments of vital signs, laboratory tests, and monitoring of adverse events. These results highlight the potential of YC-1101 as a functional ingredient in nutritional supplements to improve fatigue.

## Supplemental Materials

Supplementary data for this paper are available on-line only at http://jmb.or.kr.



## Figures and Tables

**Table 1:** Composition of the YC-1101 and placebo in hard capsules.

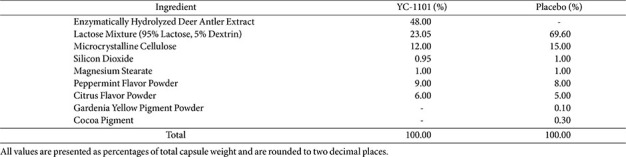

**Table 2:** Baseline characteristics of the participants^a^.

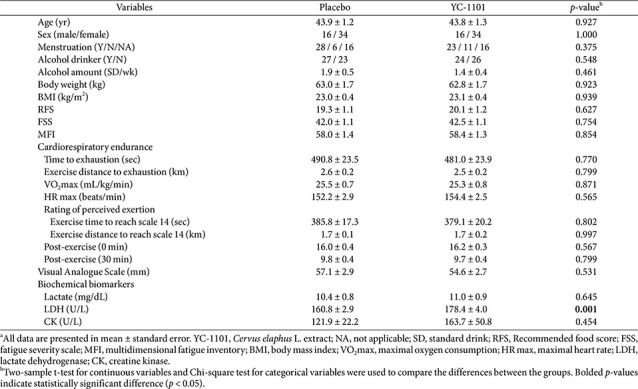

**Table 3:** Changes in multidimensional fatigue inventory scores over 8 weeks^a^

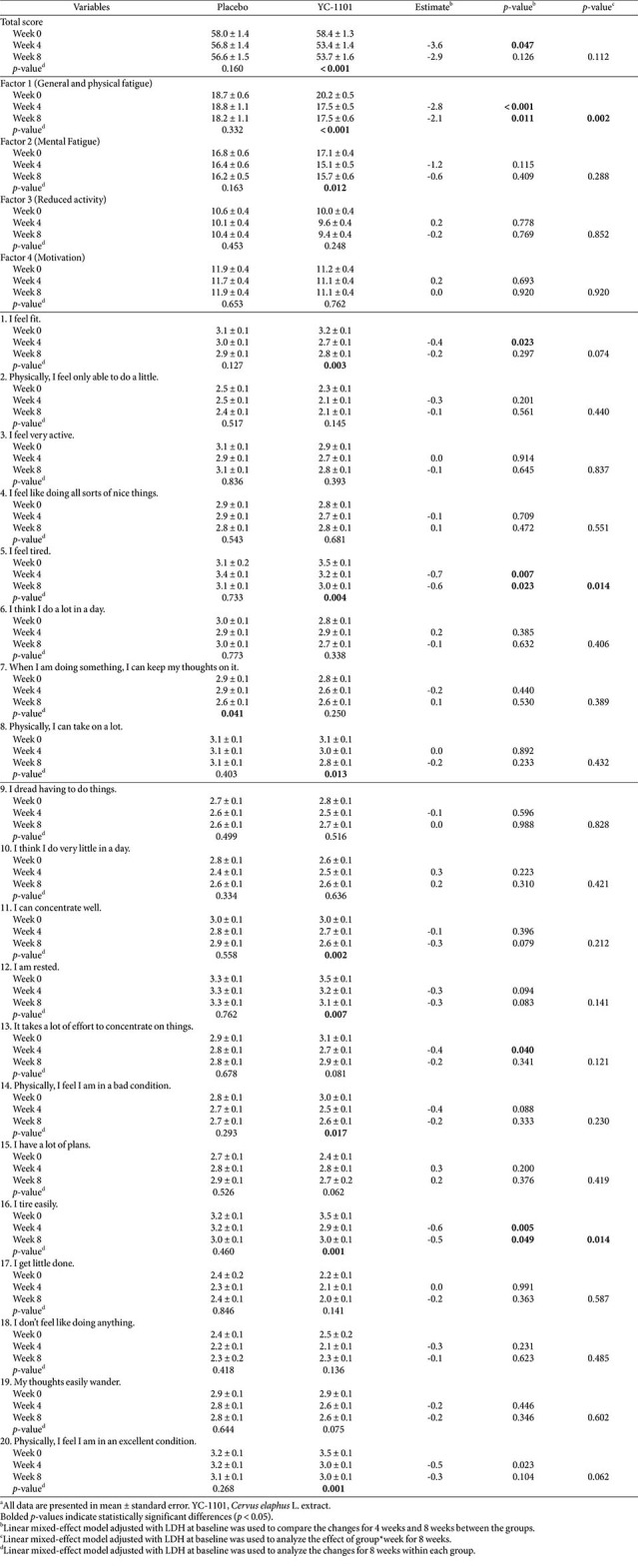

**Table 4:** Changes of fatigue severity scale (FSS) scores over 8 weeks^a^.

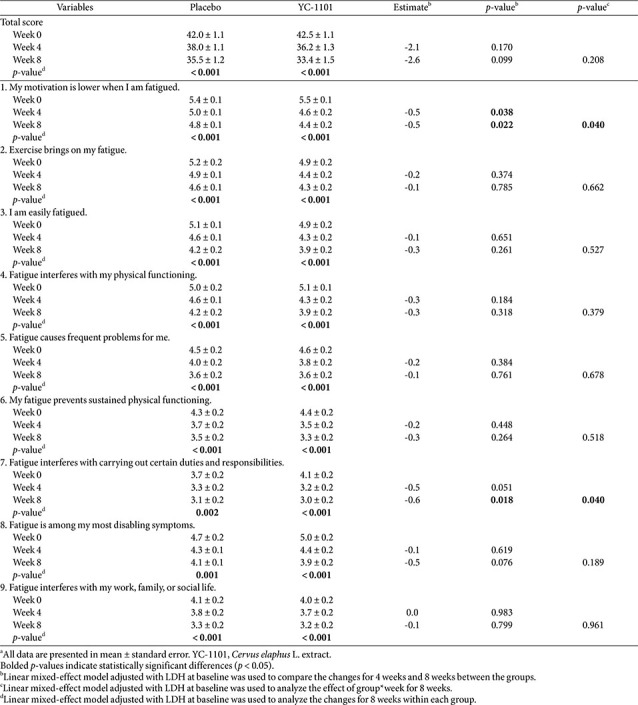

**Table 5:** Changes of blood biomarkers during recovery^a^.