# Supplementary material for: Efficacy and Safety of Enzyme-Derived Deer Velvet Extract Supplementation on Adults with Chronic Fatigue: a Randomized, Placebo-Controlled, Double-Blind Trial
Source: J Microbiol Biotechnol. 2026 Jul 9;36:e2601055. doi: 10.4014/jmb.2601.01055 (PMC13396742; doi:10.4014/jmb.2601.01055)
Supplement: Supplementary file 1 [file jmb-36-e2601055-supple.pdf]

J. Microbiol. Biotechnol. 2026. 36: e2601055

https://doi.org/10.4014/jmb.2601.01055

Table S1. Changes in dietary intake, physical activity, and sleeping time[a]

Variables	Placebo	YC-1101	*p*-value[b]
Dietary intake			
Energy (kcal/d)			
Week 0	1606 ± 56	1474 ± 47	
Week 4	1634 ± 66	1576 ± 44	
Week 8	1658 ± 63	1513 ± 43	0.202
p-value[c]	0.155	0.695	
Carbohydrate (g/d)			
Week 0	229 ± 7	209 ± 8	
Week 4	234 ± 9	225 ± 7	
Week 8	244 ± 8	215 ± 7	0.056
p-value[c]	**0.033**	0.941	
Protein (g/d)			
Week 0	64 ± 3	57 ± 2	
Week 4	64 ± 3	62 ± 2	
Week 8	66 ± 4	61 ± 2	0.564
p-value[c]	0.191	0.076	
Fat (g/d)			
Week 0	48 ± 2	46 ± 2	
Week 4	49 ± 3	48 ± 2	
Week 8	46 ± 2	46 ± 2	0.903
p-value[c]	0.597	0.919	
Sodium (mg/d)			
Week 0	3539 ± 170	3190 ± 170	
Week 4	3603 ± 192	3451 ± 157	
Week 8	3679 ± 200	3425 ± 157	0.748
p-value[c]	0.414	0.191	
Physical activity (MET-min/wk)			
Week 0	947 ± 71	1032 ± 101	
Week 4	1031 ± 86	1029 ± 99	
Week 8	948 ± 85	1083 ± 103	0.204
p-value[c]	0.751	0.456	
Sleeping time (min)			
Week 0	400.8 ± 7.4	384.0 ± 7.8	
Week 4	397.3 ± 7.3	376.9 ± 8.3	
Week 8	404.7 ± 8.1	390.5 ± 8.3	0.700
p-value[c]	0.341	0.159	

[a] All data are presented in mean ± standard error. YC-1101, *Cervus elaphus* L. extract; MET, metabolic equivalent task.

[b] Linear mixed-effect model was used to analyze the effects of group, week and group*week for 8 weeks.

[c] Linear mixed-effect model was used to analyze the changes for 8 weeks within each group.

Table S2. Changes in blood biomarkers over 8 weeks[a]

Variables	Placebo	YC-1101	*p-value*[b]
WBC (10^3/μL)			
Week 0	5.4 ± 0.2	5.3 ± 0.2	
Week 8	5.4 ± 0.2	5.6 ± 0.2	0.113
p-value[c]	0.892	**0.037**	
RBC (10^6/μL)			
Week 0	4.5 ± 0.1	4.6 ± 0.1	
Week 8	4.5 ± 0.1	4.7 ± 0.1	0.690
p-value[c]	0.258	0.574	
Hb (g/dL)			
Week 0	13.9 ± 0.2	14.2 ± 0.2	
Week 8	13.9 ± 0.3	14.1 ± 0.2	0.281
p-value[c]	0.122	0.986	
Hct (%)			
Week 0	40.8 ± 0.6	41.4 ± 0.5	
Week 8	40.8 ± 0.6	41.7 ± 0.5	0.798
p-value[c]	0.543	0.337	
PLT (10^3/μL)			
Week 0	249.7 ± 6.4	247.2 ± 7.2	
Week 8	254.8 ± 7.6	250.8 ± 8.1	0.789
p-value[c]	0.384	0.217	
MCV (fL)			
Week 0	90.4 ± 0.7	89.8 ± 0.6	
Week 8	90.1 ± 0.8	89.8 ± 0.7	0.051
p-value[c]	0.129	0.207	
MCH (pg)			
Week 0	30.7 ± 0.3	30.7 ± 0.3	
Week 8	30.6 ± 0.4	30.5 ± 0.3	0.619
p-value[c]	0.909	0.417	
MCHC (g/dL)			
Week 0	34.0 ± 0.2	34.2 ± 0.2	
Week 8	33.9 ± 0.2	33.9 ± 0.2	0.247
p-value[c]	0.824	0.160	
Neutrophil (%)			
Week 0	55.3 ± 1.1	52.1 ± 1.5	
Week 8	54.2 ± 1.1	53.2 ± 1.3	0.186
p-value[c]	0.320	0.377	

Table S2. Changes in blood biomarkers over 8 weeks[a] (continued)

Variables	Placebo	YC-1101	*p-value*[b]
Lymphocyte (%)			
Week 0	35.1 ± 1.1	36.9 ± 1.3	
Week 8	36.1 ± 1.1	36.6 ± 1.2	0.390
p-value[c]	0.309	0.840	
Monocyte (%)			
Week 0	7.1 ± 0.3	7.5 ± 0.3	
Week 8	7.2 ± 0.3	7.2 ± 0.2	0.270
p-value[c]	0.705	0.239	
Eosinophil (%)			
Week 0	2.1 ± 0.1	3.1 ± 0.5	
Week 8	2.2 ± 0.2	2.6 ± 0.3	0.052
p-value[c]	0.809	**0.013**	
Basophil (%)			
Week 0	0.4 ± 0.0	0.4 ± 0.0	
Week 8	0.4 ± 0.0	0.4 ± 0.0	0.319
p-value[c]	0.211	0.871	
ALT (U/L)			
Week 0	17.3 ± 1.6	18.1 ± 1.4	
Week 8	17.6 ± 1.4	17.0 ± 1.3	0.305
p-value[c]	0.963	0.164	
AST (U/L)			
Week 0	21.0 ± 1.6	22.3 ± 1.3	
Week 8	21.7 ± 2.0	22.4 ± 1.5	0.978
p-value[c]	0.500	0.529	
ALP (U/L)			
Week 0	60.2 ± 2.1	63.2 ± 2.5	
Week 8	61.5 ± 2.3	63.5 ± 2.7	0.417
p-value[c]	0.116	0.675	
BUN (mg/dL)			
Week 0	13.1 ± 0.5	13.0 ± 0.5	
Week 8	13.1 ± 0.5	12.5 ± 0.4	0.261
p-value[c]	0.541	0.328	
Creatinine (mg/dL)			
Week 0	0.77 ± 0.02	0.79 ± 0.02	
Week 8	0.77 ± 0.02	0.79 ± 0.03	0.227
p-value[c]	0.085	0.991	

Table S2. Changes in blood biomarkers over 8 weeks[a] (continued)

Variables	Placebo	YC-1101	*p*-value[b]
Total bilirubin (mg/dL)			
Week 0	0.58 ± 0.04	0.63 ± 0.04	
Week 8	0.59 ± 0.04	0.61 ± 0.04	0.582
p-value[c]	0.680	0.713	
Uric acid (mg/dL)			
Week 0	4.6 ± 0.2	4.9 ± 0.2	
Week 8	4.4 ± 0.2	4.9 ± 0.2	0.635
p-value[c]	0.529	0.965	
Total protein (g/dL)			
Week 0	7.2 ± 0.1	7.3 ± 0.0	
Week 8	7.3 ± 0.0	7.2 ± 0.1	0.134
p-value[c]	0.181	0.428	
Albumin (g/dL)			
Week 0	4.6 ± 0.0	4.7 ± 0.0	
Week 8	4.6 ± 0.0	4.6 ± 0.0	0.385
p-value[c]	0.524	0.066	
Glucose (mg/dL)			
Week 0	95.8 ± 1.7	94.1 ± 1.2	
Week 8	94.7 ± 1.6	94.0 ± 1.4	0.565
p-value[c]	0.319	0.859	

[a] All data are presented in mean ± standard error. YC-1101, *Cervus elaphus* L. extract; WBC, white blood cell; RBC, red blood cell; Hb, hemoglobin; Hct, hematocrit; PLT, platelet; MCV, mean corpuscular volume; MCH, mean corpuscular hemoglobin; MCHC, mean corpuscular hemoglobin concentration; ALT, alanine aminotransferase; AST, aspartate aminotransferase; ALP, alkaline phosphatase; BUN, blood urea nitrogen. Bolded *p-value* indicate statistically significant difference ($p < 0.05$).
[b] Linear mixed-effect model was used to analyze the effects of group, week and group*week for 8 weeks.
[c] Linear mixed-effect model was used to analyze the changes for 8 weeks within each group.

Table S3. Changes in urine biomarkers over 8 weeks[a]

Variables	Placebo		YC-1101		*p*-value[b]
	NCS	CS	NCS	CS	
pH					
Week 0	50	0	50	0	-
Week 8	45	0	44	0	-
p-value[c]	-		-		
Protein					
Week 0	50	0	50	0	-
Week 8	45	0	44	0	-
p-value[c]	-		-		
Glucose					
Week 0	50	0	50	0	-
Week 8	45	0	44	0	-
p-value[c]	-		-		
Ketone					
Week 0	50	0	50	0	-
Week 8	45	0	44	0	-
p-value[c]	-		-		
Urobilinogen					
Week 0	50	0	50	0	-
Week 8	45	0	44	0	-
p-value[c]	-		-		
Bilirubin					
Week 0	50	0	50	0	-
Week 8	45	0	44	0	-
p-value[c]	-		-		
Nitrite					
Week 0	50	0	50	0	-
Week 8	45	0	44	0	-
p-value[c]	-		-		
Specific gravity					
Week 0	50	0	50	0	-
Week 8	45	0	44	0	-
p-value[c]	-		-		

[a] Number of subjects. YC-1101, *Cervus elaphus* L. extract; NCS, not clinically significant; CS, clinically significant.

[b] Fishers' exact test was used to compare the difference between the groups and *p*-values were not computed because CS column contains all zeros.

[c] McNemar's test was used to compare the difference within each group and *p*-values were not computed because CS column contains all zeros.

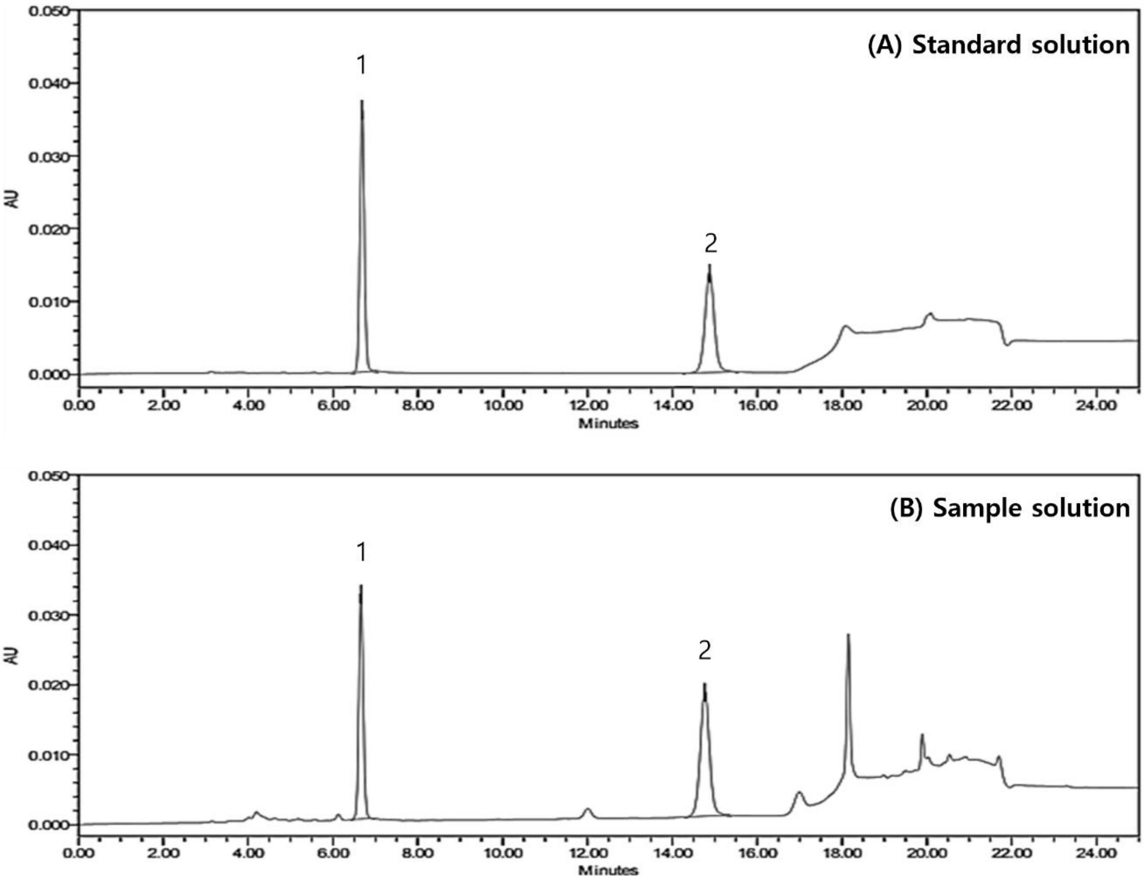

Fig. S1. HPLC chromatograms of YC-1101. (A) Standard solution containing uracil and hypoxanthine. **(B)** YC-1101 sample solution. Peak 1: uracil (RT 6.7 min); Peak 2: hypoxanthine (RT 14.8 min).
